# Marsupialization followed by enucleation of a large maxillary dentigerous cyst in a young child: A case report and literature review

**DOI:** 10.1016/j.ijscr.2024.110346

**Published:** 2024-09-25

**Authors:** Toan Van Phan, Dat Gia Phan, Hoang Minh Phan, Hien Minh Nguyen

**Affiliations:** a Oral Biology International Program, Faculty of Dentistry, Chulalongkorn University, Bangkok, Thailand; b Oral Surgery Residency Program, Faculty of Dentistry, University of Medicine and Pharmacy, Ho Chi Minh city, Viet Nam; c Hospital for Rehabilitation - Professional Diseases, Ho Chi Minh city, Viet Nam

**Keywords:** Case report, Child, Dentigerous cyst, Marsupialization

## Abstract

**Introduction:**

This report presents a rare case of a dentigerous cyst (DC) associated with the maxillary right canine in a young child, successfully treated with marsupialization followed by enucleation.

**Presentation of case:**

A 9-year-old girl was referred to a maxillofacial hospital after routine dental exam revealed a large lesion in maxilla. Radiograph showed a 5 × 6 cm unilocular radiolucent lesion in the right maxillary sinus, with destruction of the sinus and nasal cavity walls and displacement of the upper right canine into the floor of the right eye. Marsupialization was performed, and the patient was monitored every 6 months. Histology confirmed a dentigerous cyst lined with non-keratinized stratified cuboidal squamous epithelium. One-year post-surgery, the cyst had significantly reduced in size, and the canine had descended. A second surgery was performed to completely remove the cyst and the associated canine.

**Discussion:**

This case demonstrates the successful management of a large dentigerous cyst in a 9-year-old patient through staged treatment, initially with marsupialization, followed by complete cyst removal. The conservative approach allowed for the reduction of the cyst size and facilitated the natural descent of the displaced canine. Regular follow-up and timely surgical intervention were crucial in achieving a positive outcome and preventing recurrence.

**Conclusion:**

This case highlights the effectiveness of marsupialization in managing maxillary dentigerous cysts in young children.

## Highlights

• A large dentigerous cyst was found in a 9-year-old's maxilla, an unusual site and age for this condition.
• Marsupialization reduced the cyst's size and allowed the displaced canine to descend, demonstrating the benefits of conservative management.
• After one year, the cyst shrank, bone regenerated, and the canine was repositioned, leading to effective secondary cyst removal.
• Marsupialization followed by enucleation is a viable approach for large dentigerous cysts in children, preserving structures and avoiding more invasive procedures.

## 1 Introduction

A dentigerous cyst (DC) is an odontogenic cyst originating from the dental follicle of an unerupted tooth. They are the second most common type of odontogenic cyst, comprising 11.4 % to 35.5 % of such cysts, second only to radicular cysts [1]. DCs are most frequently diagnosed in individuals in their twenties to thirties, with a slight male predominance and no significant ethnic variation [2]. These cysts develop in 2.6 % to 4 % of individuals with impacted teeth, with 95 % of cases associated with permanent teeth and 5 % with supernumerary teeth [3,4]. The majority of DC (71.5 %) are located in the mandible, while the remaining cases occur in the maxilla, most commonly involving the maxillary canines [5].

Clinically, DC are often asymptomatic unless they become infected. Occasionally, swelling of the cortical bone in the maxilla or mandible is observed. DC are usually discovered incidentally on radiographs taken for unrelated purposes, such as routine examinations, orthodontic treatment, or evaluation of an unerupted tooth [6]. Radiographically, DC usually appear as well-defined, unilocular radiolucent lesions with a homogeneous appearance and a round or ovoid shape [3]. These cysts are commonly attached to the cementoenamel junction of an unerupted tooth. The borders of DC can be sclerotic, although they become less distinct if the cyst becomes infected [7]. The gradual expansion of DC can displace dental roots and nerves, though the bone cortex generally remains intact [8].

The histopathologic characteristics of DC differ based on the cyst's inflammatory state. In non-inflamed cysts, the epithelial lining comprises 2–4 layers of stratified nonkeratinizing cells, with the underlying connective tissue being loose [9]. In inflamed cysts, the connective tissue is infiltrated by inflammatory cells, resulting in a thicker epithelial lining that may develop hyperplastic rete ridges [10].

The common treatment for DC is enucleation, which entails the complete removal of the cyst and the associated tooth [11]. For larger cysts, marsupialization followed by enucleation is an alternative option when enucleation could potentially damage surrounding tissues, such as nerves, or cause bone fractures [11]. This study presents a case of a young girl with a large dentigerous cyst associated with a maxillary canine, successfully treated through marsupialization followed by enucleation.

## 2 Presentation of case

A 9-year-old girl was referred to a maxillofacial hospital due to a large lesion discovered on a panoramic radiograph during a routine dental examination. During the extraoral examination, a slight swelling was observed at the right nasolabial fold, with the overlying skin appearing normal. The patient reported no pain or numbness in the facial skin, and her eye movement was normal. The intraoral examination revealed a soft, compressible expansion in the right maxillary vestibule, extending from the upper right central incisor to the upper right first molar, measuring approximately 1 × 5 cm. The overlying mucosa appeared normal, with no signs of inflammation or fistula. The dental examination showed that the upper right lateral incisor had not erupted, the primary upper right canine was mobile at level 2, and the primary upper right first and second molars were decayed, with half of the crowns missing.

**Fig. 1:**
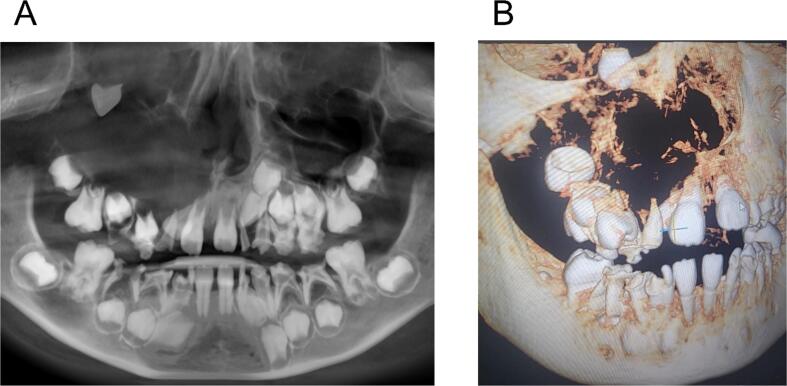
(A) Panoramic graph showed a radiolucent lesion in the right maxilla. The size of the lesion was about 5 × 6 cm. Upper right canine was displaced into the floor of the right eye. (B) Three-dimensional (3D) rendering image revealed the destruction of the lateral wall of nasal cavity and the walls of right maxillary sinus.

Cone beam computed tomography (CBCT) radiograph revealed a unilocular radiolucent lesion in the right maxillary sinus (Fig. 1). The lesion measured approximately 5 × 6 cm and had destroyed the lateral wall of the right maxillary sinus and nasal cavity. The upper right canine had been displaced into the floor of the right eye. Given the large size of the cyst and the potential for enucleation to damage surrounding structures, including the eye and nasal cavity, we opted for a marsupialization procedure followed by enucleation.

**Fig. 2:**
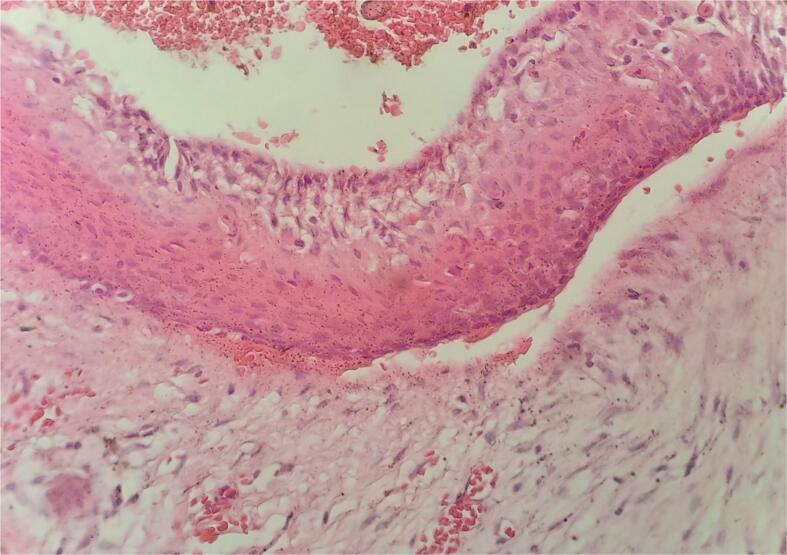
Hematoxylin and eosin staining of cyst membrane specimen showed a non-keratinized stratified cuboidal squamous epithelium.

The surgery was performed under local anesthesia. A buccal mucoperiosteal flap was elevated to access the cyst membrane. The cyst membrane was then fenestrated, and a trimmed endotracheal tube, measuring 1 cm in diameter and 4 cm in length, was placed into the cyst. This tube was secured to the upper right central incisor using stainless steel wire. The patient's parents were instructed to assist her in irrigating the cyst with normal saline three times a day. A sample of the cyst was collected and sent to the oral pathology department for histological evaluation. Histology revealed that the cyst was lined with non-keratinized stratified cuboidal squamous epithelium (Fig. 2). The underlying connective tissue was loose, with no signs of inflammatory cell infiltration. Radiographic and histological findings confirmed the lesion as a dentigerous cyst.

**Fig. 3:**
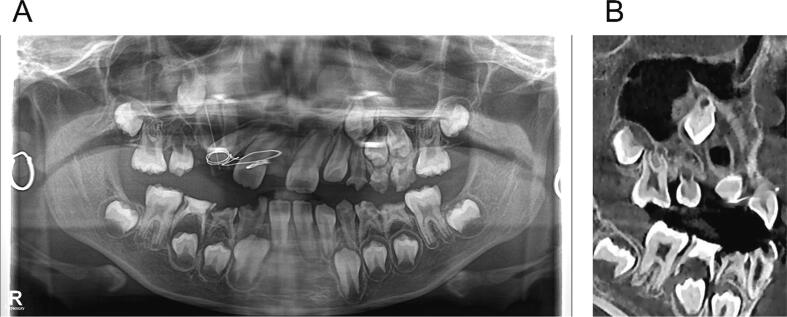
(A) Panoramic graph after 1 year of marsupialization showed the upper right canine had been pulled down, and the lateral wall of the nasal cavity had regenerated. (B) Sagittal plane of CBCT demonstrated the bone formation inside the cyst cavity.

The patient was scheduled for radiographs every six months. After one year, the radiograph showed that the cyst had shrunk, the upper right canine had been pulled down, and the lateral wall of the nasal cavity had regenerated (Fig. 3).

**Fig. 4:**
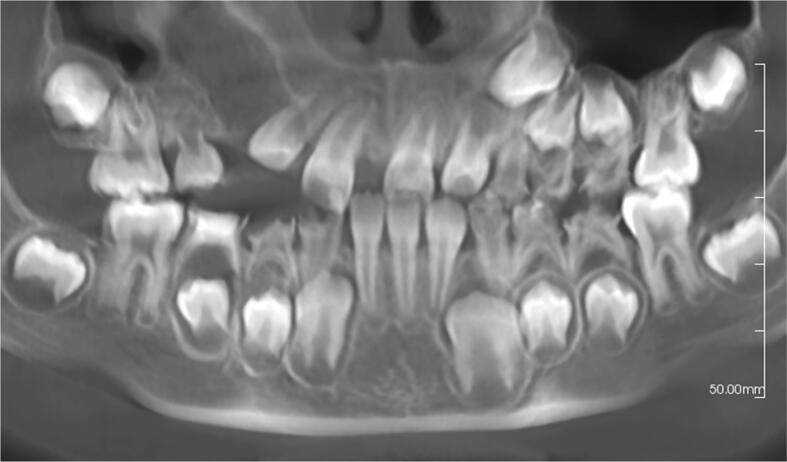
Panoramic radiograph after cyst enucleation showed the removal of the cyst and associated upper right canine.

The patient then underwent a second surgery to completely remove the cyst and the related upper right canine (Fig. 4). Subsequently, the patient was referred to the orthodontic department for further treatment planning. This work has been reported in line with the SCARE 2023 criteria [11].

## 3 Discussion

A dentigerous cyst is the second most common odontogenic cyst after the radicular cyst, typically associated with the crown of an impacted or unerupted tooth [12]. DC are generally asymptomatic and often discovered through radiographs, with the mandibular third molar being the most commonly affected tooth. These cysts usually occur in individuals aged 20–30 [13]. This report, however, presents a case of a dentigerous cyst occurring in a young child and involving the maxilla.

Literature review identified only 10 reported cases of DC in children under 10 years old, specifically involving the maxilla, in the English literature (Table 1). Available data on age and gender showed the youngest patient was 4 years old, with 8 out of 10 cases occurring in male patients. Anatomical analysis revealed that 8 cases were located in the incisor and canine regions. In terms of treatment, marsupialization or decompression was used in 8 cases, while enucleation was performed in 2 cases.

**Table 1:** Literature review.

Author	Year	Age	Gender	Location	Treatment	Reference
Sain	1992	9	Female	Maxillary right canine	Marsupialization	[14]
Kalaskar	2007	7	Male	Maxillary right central incisor	Enucleation	[15]
Lee	2004	10	Male	Maxillary right sinus	Marsupialization	[16]
Gondim	2008	4	Male	Maxillary right central incisor	Marsupialization	[17]
Pramod	2011	6	Male	Maxillary left canine	Enucleation	[18]
Sasaki	2012	10	Male	Maxillary second premolar	Marsupialization	[19]
Bowdin	2021	7	Male	Maxillary central incisors	Marsupialization	[20]
Inturi	2022	10	Male	Maxillary left canine	Decompression	[21]
Khalifa	2023	7	Female	Maxillary left canine	Marsupialization	[22]
Antunes	2023	10	Male	Maxillary left lateral incisor and canine	Marsupialization	[23]

Marsupialization is a surgical technique used to treat DC, offering several advantages, including the reduction of the cyst cavity and the preservation of the involved structures. This method involves creating a surgical opening in the cyst and suturing its edges to the surrounding mucosa, which allows the cyst to remain open and drain [24]. A drainage tube can be inserted into the cyst cavity to ensure effective irrigation and prevent the opening from closing [25]. Over time, the cyst cavity reduces in size, potentially facilitating the natural eruption of the tooth. One of the primary benefits of marsupialization is that it can preserve the tooth associated with the cyst, which is particularly advantageous for young patients whose teeth are still developing. However, a significant limitation is the unpredictable nature of tooth eruption following marsupialization [26]. The decision to use this method can be complicated by the lack of robust clinical evidence on the likelihood of tooth eruption post-treatment. Consequently, many clinicians may opt for complete enucleation and tooth extraction instead. Nonetheless, studies have demonstrated the effectiveness of marsupialization under certain conditions.

Factors such as tooth inclination, root maturity, and the availability of space in the alveolar bone serve as predictive indicators for successful tooth eruption. For instance, Gondim et al. documented a case where a dentigerous cyst associated with the germ of a permanent maxillary central incisor in a 4-year-old boy developed due to trauma from the primary incisor [17]. This cyst was effectively treated with marsupialization. After 36 months of follow-up, the permanent incisor erupted in its correct position in the oral cavity without the need for orthodontic traction. In our case, we opted to completely remove the cyst and the involved tooth because we discovered bone formation inside the cyst cavity, which could potentially lead to future impaction of the canine.

This case report provides evidence of the successful marsupialization of a large dentigerous cyst in the maxilla, particularly in young children.

## Consent

Written informed consent was obtained from the patient's parents/legal guardian for publication and any accompanying images. A copy of the written consent is available for review by the Editor-in-Chief of this journal on request.

## Ethical approval

Because this is a single case report, the Institutional Review Board at Hospital for rehabilitation - professional diseases, Ho Chi Minh city, Vietnam waived the need for ethical approval.

## Funding

This case report did not receive any specific grant from funding agencies in the public, commercial or not-for-profit sectors.

## Guarantor

The guarantors for this study are T.V.P. and H.M.N., who accept full responsibility for the work, the conduct of the study, had access to the data, and controlled the decision to publish.

## Credit authorship contribution statement

T.V.P. contributed to conception, data collection, and writing manuscript draft. D.G.P. contributed to data collection, and revising manuscript draft. H.M.P. contributed to revising manuscript draft. H.M.N. contributed to conception, design, editing, writing, and revising manuscript draft. All authors gave their final approval and agreed to be accountable for all aspects of the work.

## Declaration of competing interest

We declare there are no conflicts of interest.
